# Simvastatin preparations promote PDGF‐BB secretion to repair LPS‐induced endothelial injury through the PDGFRβ/PI3K/Akt/IQGAP1 signalling pathway

**DOI:** 10.1111/jcmm.14709

**Published:** 2019-10-01

**Authors:** Xia Zheng, Wang Zhang, Zhen Wang

**Affiliations:** ^1^ Department of Critical Care Medicine The First Affiliated Hospital, College of Medicine Zhejiang University Hangzhou China; ^2^ Department of Infectious Diseases Sir Run Run Shaw Hospital, College of Medicine Zhejiang University Hangzhou China; ^3^ Department of Cardiology The First Affiliated Hospital, College of Medicine Zhejiang University Hangzhou China

**Keywords:** endothelial injury, IQ‐GTPase‐activating protein, nanoparticle, platelet‐derived growth factor receptor, simvastatin

## Abstract

Endothelial barrier dysfunction is a critical pathophysiological process of sepsis. Impaired endothelial cell migration is one of the main reasons for endothelial dysfunction. Statins may have a protective effect on endothelial barrier function. However, the effect and mechanism of statins on lipopolysaccharide (LPS)‐induced endothelial barrier dysfunction remain unclear. Simvastatin (SV) was loaded in nanostructured lipid carriers to produce SV nanoparticles (SV‐NPs). Normal SV and SV‐NPs were used to treat human umbilical vein vascular endothelial cells (HUVECs) injured by LPS. Barrier function was evaluated by monitoring cell monolayer permeability and transendothelial electrical resistance, and cell migration ability was measured by a wound healing assay. LY294002 and imatinib were used to inhibit the activity of PI3K/Akt and platelet‐derived growth factor receptor (PDGFR) β. IQ‐GTPase‐activating protein 1 (IQGAP1) siRNA was used to knockdown endogenous IQGAP1, which was used to verify the role of the PDGFRβ/PI3K/Akt/IQGAP1 pathway in SV/SV‐NPs‐mediated barrier protection in HUVECs injured by LPS. The results show that SV/SV‐NPs promoted the migration and decreased the permeability of HUVECs treated with LPS, and the efficacy of the SV‐NPs exceeded that of SV significantly. LY294002, imatinib and IQGAP1 siRNA all suppressed the barrier protection of SV/SV‐NPs. SV/SV‐NPs promoted the secretion of platelet‐derived growth factor‐BB (PDGF‐BB) and activated the PDGFRβ/PI3K/Akt/IQGAP1 pathway. SV preparations restored endothelial barrier function by restoring endothelial cell migration, which is involved in the regulation of the PDGFRβ/PI3K/Akt/IQGAP1 pathway and PDGF‐BB secretion. As an appropriate formulation for restoring endothelial dysfunction, SV‐NPs may be more effective than SV.

## 1 INTRODUCTION

Sepsis is a life‐threatening organ dysfunction resulting from a dysregulated host response to infection,^1^ which is a major cause of death in critically ill patients.^2^ Microcirculatory dysfunction, which involves a variety of mechanisms, is thought to be a key pathophysiological process in sepsis. Functional changes resulting from vascular endothelial cell injury play an important role in the pathogenesis of sepsis. Increased vascular permeability, resulting in severe fluid exudation, is an important mechanism in vascular endothelial injury, leading to microcirculation disturbances. In this process, the inflammatory responses of the vascular endothelium and vascular leakage provide areas for the entry of innate immune cells and humoural immunity‐affecting molecules.^3^, ^4^ However, severe exudation also leads to serious organ damage, including acute lung injury (ALI)/acute respiratory distress syndrome (ARDS) and acute kidney injury (AKI).^5^

Cytoskeletal rearrangements and related changes in cell migration are a major cause of increased endothelial cell permeability. A variety of proteins and cellular components is involved in the regulation of the cytoskeleton. Among them, IQ‐GTPase‐activating protein 1 (IQGAP1) has become a key component of cell cytoskeletal dynamic regulation during cell migration, maintaining cell‐cell connections, microbial pathogenic mechanisms and intracellular substance transport.^6^, ^7^, ^8^, ^9^ IQGAP1, identified in 1994, is a widely expressed protein containing IQ motifs with a molecular weight of 190 kD.^10^ Recent studies have shown that IQGAP1 is involved in the regulation of endothelial barrier function. Silencing IQGAP1 in human microvascular endothelial cells resulted in the disruption of adherens junctions, the formation of interendothelial gaps and a reduction in barrier function.^11^ Another study also found that IQGAP1 was an important factor in regulating vascular endothelial cell permeability during acute lung injury caused by endotoxins and bacteria.^6^ IQGAP1 was identified as a scaffold protein that regulates the actin cytoskeleton alone or with its binding partners,^12^ including Akt and extracellular signal‐regulated kinase, which are involved in cell migration and proliferation.^13^, ^14^ Previous studies have also reported that IQGAP1 interacts with growth factors such as vascular endothelial growth factor receptor 2 (VEGFR2) and platelet‐derived growth factor receptor (PDGFR), and by this may interfere with receptor's signalling leading to an altered actin cytoskeleton and cell motility.^15^, ^16^ It has been reported that PDGFRβ signalling plays an active role in blood vessel and post‐injury tissue recovery^17^ and that the overexpression of PDGFRβ in endothelial progenitor cells promotes vascular repair in the early phase after vascular injury through enhanced cell proliferation, migration and angiogenesis.^18^, ^19^ However, the relationship between IQGAP1 and PDGF/PDGFR signalling in endothelial cell migration after vascular injury is unknown.

Numerous animal experiments and cell studies have shown that statins have a positive effect on vascular injury in sepsis by reducing inducible nitric oxide (NO) synthase‐mediated NO production and by inhibiting cytokines and their downstream signal transduction pathways.^20^, ^21^, ^22^, ^23^ Other studies have reported that statins failed to affect the prognosis of sepsis^24^, ^25^ and increased doses raised the risk of liver and kidney damage.^26^ However, a recent editorial noted that the cause of some heterogeneity among previous findings was unclear and that most reports were retrospective studies, which were not well randomized.^27^ The clinical application of statins to sepsis patients is presently controversial. Simvastatin nanoparticles (SV‐NPs), a tool for the targeted delivery of SV using nanostructured lipid carriers, are an improved formulation that facilitate the drug's protective effects on endothelial or other cells without increasing the dose and avoiding unnecessary liver and kidney damage.^28^, ^29^, ^30^ This study therefore investigated whether different SV preparations had protective effects on the vascular endothelium and whether SV preparations repaired LPS‐injured endothelial cells through the IQGAP1/PDGFRβ‐binding complex and related signalling pathways.

## 2 MATERIALS AND METHODS

### 2.1 Cell culture

Human umbilical vein vascular endothelial cells isolated from human umbilical vein vascular (Catalog Number: 8000, ScienCell, San Diego, California) were cultivated by Endothelial Cell Medium (ECM, Cat. No: 1001, ScienCell) with 5% of foetal bovine serum (FBS, Cat. No: 0025, ScienCell), 1% endothelial cell‐derived growth factor (ECGS, Cat. No: 1052, ScienCell) and 1% penicillin/streptomycin (P/S, Cat. No: 0503, ScienCell) and were incubated in 37°C and 5% CO_2_. LPS (LPS from Escherichia coli 055: B5, Cat. No: L2880, Sigma, Germany), simvastatin (Cat. No: S6196, Sigma, Germany), PI3K inhibitor LY294002 (#9901, Cell Signaling Technology, USA) and PDGFRβ inhibitor Imatinib (Cat. No: STI571, Selleck, USA) were attenuated by ECM without FBS, and HUVECs were interfered for 24 hours with LPS doses of 10 μg/mL, and SV/SV‐NPs were used at the concentration of 1 μmol/L, LY294002 was used at the concentration of 25 μmol/L, and Imatinib was used at the concentration of 20 μmol/L.

### 2.2 Preparation of simvastatin‐loaded NLCs

The simvastatin nanoparticles (SV‐NPs), a new formulation of simvastatin using nanostructured lipid carriers, were prepared by solvent diffusion method in an aqueous system as described previously.^31^, ^32^ Briefly, 97.5 mg monostearin (Shanghai Chemical Reagent Co., Ltd. Shanghai, China), 30 mg Medium‐chain triglycerides (MCT, Gattefosse, Saint‐Priest, France), 15 mg PEG2000‐stearic acid (Tokyo Kasei Kogyo Co., Ltd., Tokyo, Japan) and 7.5 mg simvastatin were completely dissolved into 0.75 mL ethanol in water bath at 60°C. Then, 0.025 mL solution was diluted to 0.25 mL by ethanol. The resultant organic solution was quickly dispersed into 4.75 mL distilled water under mechanical agitate at 400 rpm in water bath at 60°C for 0.5 minutes. The SV‐NPs with 1 mg/mL concentration were prepared, respectively.

### 2.3 Small interfering RNA (siRNA) transfections

Control siRNA and IQGAP1‐specific siRNA were obtained from QIAGEN (Duesseldorf, Germany), and siRNA was transfected into HUVECs at 30 pmol per 1.0 × 10^5^ cells using INTERFERin (Cat. No: 409‐10; Polyplus, NY, USA) according to the manufacturer's protocol. Six hours after transfection, culture medium was replaced by an equal amount of 1% serum ECM and then treated for 24 hours.

### 2.4 Transwell‐Evans blue monolayer permeability assay

Transwell inserts (pore size 3.0 μm, #3472, Costar, USA) were used to evaluate permeability as described previously.^33^ In brief, HUVECs were seeded in Transwell inserts at the concentration of 1 × 10^4^ cells/well and incubated in 37°C and 5% CO_2_ for 48 hours, and then, cells were treated by LPS, SV/SV‐NP, LY294002 or Imatinib. Evans blue (EB, Cat. No: E2129, Sigma, Germany)‐conjugated albumin (final concentration: 0.67 mg/mL) was prepared by diluting a stock solution of 2% EB in a 60‐fold excess of bovine serum albumin (BSA, 4%) in order to eliminate any free EB, according to a method previously described.^33^ After that, culture medium in the upper chamber was removed and 100 μL EB‐conjugated albumin was added to the upper chamber, and 500 μL 4% BSA was added to the lower chamber. Moreover, the heights of the EB‐conjugated albumin in the upper and 4% BSA in the lower compartments were at the same level in order to eliminate influence of hydrostatic pressure gradient. After incubated for 1 hour at 37°C in 5% CO_2_, the mixture in the lower chamber was collected. Finally, the absorbance was determined at 620 nm wavelength by a micro‐plate reader. Calculated EB‐albumin leak trans‐HUVECs according to standard curve.

### 2.5 Transendothelial electric resistance measurements

Endothelial barrier integrity was determined using by the electrical resistance system (Millicell‐ERS, MERS00002; Merck Millipore, Germany). Cells were treated as we described previously, and then, electric resistance of monolayer HUVECs was measured every 4 hours; Transendothelial electric resistance (TEER) was calculated based on the manufacturer's recommendations. Then, TEER was graphed at indicated time‐points during an experiment at 24 hours. A higher TEER represented a higher barrier integrity.

### 2.6 Wound healing assay

Based on previous description,^34^ HUVECs were seeded in 6‐well plates. Confluent monolayer cells were scraped by using a 1000 μL pipette tip and then washed with phosphate‐buffered saline (PBS) to clear cell debris and suspension. Replace complete medium with 1% serum ECM, and cells were treated separately and then incubated for 24 hours at 37°C in 5% CO_2_. The images were captured under a microscope at the 0 hour and 24 hours at the same position of the wound. The migration ability was measured by the rates of scratch wound confluence using Adobe Photoshop 2017 software (Adobe Systems Inc, San Jose, CA, USA).

### 2.7 Immunofluorescence staining

The HUVECs were treated as we described previously.^34^ Cell was incubated with FITC‐phalloidin (Cat. No: P5282; Sigma, Germany), VE‐Cadherin Rabbit antibody (1:400, #2500; Cell Signal Technology, USA) and Claudin‐5 Rabbit antibody (1:200, Cat. No. ab15106; Abcam, Cambridge, MA, USA) overnight at 4°C. After washing three times, the cells were incubated with CoraLite594‐conjugated Goat Anti‐Rabbit IgG(H+L) (1:200, Cat. No. SA00013‐4; Proteintech) and CoraLite488–conjugated Affinipure Goat Anti‐Rabbit IgG(H+L) (1:200, Cat. No. SA00013‐2; Proteintech) for 1 hour. The HUVECs were again washed with PBS and incubated with DAPI (1:2000, Cat. No. D9564; Sigma‐Aldrich) for 10 minutes. Finally, the cells were washed three times again in PBS and observed under a confocal microscope (Olympus FV‐1000).

### 2.8 Western blot

Total proteins of HUVECs were extracted as described previously,^34^ the protein concentrations were measured using a BCA Protein Quantification Kit (Cat. No. 23227; Thermo Fisher Scientific, Waltham, MA, USA), and protein samples were thereafter boiled with 5× loading buffer (Cat. No. P0015L, Beyotime), then separated by SDS‐PAGE, transferred to the polyvinylidene difluoride (PVDF) membrane (Cat. No. IPVH00010; Merck Millipore, Germany) and then incubated in 5% skimmed milk for 1 hour at room temperature. Following incubated with primary antibodies at different concentration at 4°C overnight, including IQGAP1 (1:1000, #29016, Cell Signal Technology, USA), p‐PDGFRβ (1:1000, #2227, Cell Signal Technology, USA), PDGFRβ (1:1000, #3169, Cell Signal Technology, USA), Akt (1:1000, #4685, Cell Signal Technology, USA), p‐Akt (1:2000, #4060, Cell Signal Technology, USA) and GAPDH (1:1000, #5174, Cell Signal Technology, USA), the membrane was incubated with a horseradish peroxidase (HRP)‐conjugated secondary antibody and developed by enhanced chemiluminescence kit (Cat. No. 70‐P1421; MultiSciences Biotech, Hangzhou, China) and exposed to X‐ray film finally.

### 2.9 Co‐Immunoprecipitation

Cells were lysed with 250 μL of ice‐cold lysis buffer as described before. For immunoprecipitation, 20 μL of protein A magnetic beads (#161‐4013, BIO‐RAD, USA) was precipitated with IQGAP1 antibody (1:100, #29016, Cell Signal Technology, USA) for 1 hour at room temperature. After incubating with IQGAP1‐protein A magnetic beads overnight at 4°C, IP controls were realized, in same conditions, with a normal rabbit IgG (Cat. No. A7016, Beyotime). The beads were washed and then boiled with loading buffer, transferred to immunoblots and incubated with IQGAP1 (1:1000, #29016, Cell Signal Technology, USA) and PDGFRβ (1:1000, #3169, Cell Signal Technology, USA) antibodies, and then, the association between IQGAP1 and PDGFRβ was quantified. The ratio of PDGFRβ (IP) to IQGAP1 (IP) represented the binding ability of PDGFRβ/IQGAP1.

### 2.10 ELISA

Culture medium was removed after treated and stored at −80°C. The levels of PDGF‐BB in supernatants of the cultures were determined using ELISA kits (Cat. No. 70‐EK91372; MultiSciences Biotech, China) based on the manufacturer's recommendations.

### 2.11 Statistical analysis

All data were expressed as mean ± standard deviation (SD). One‐way analysis of variance (ANOVA) was used for multiple‐group comparisons. GraphPad Prism 7.0 (GraphPad software Inc, San Diego, CA, USA) was used for analysis. The difference was statistically significant at *P* < .05.

## 3 RESULTS

### 3.1 Simvastatin preparations reduced the high‐permeability of HUVEC monolayers, increased the low‐migration ability induced by LPS, remodelled the cytoskeleton and improved endothelial barrier function

**Figure 1:**
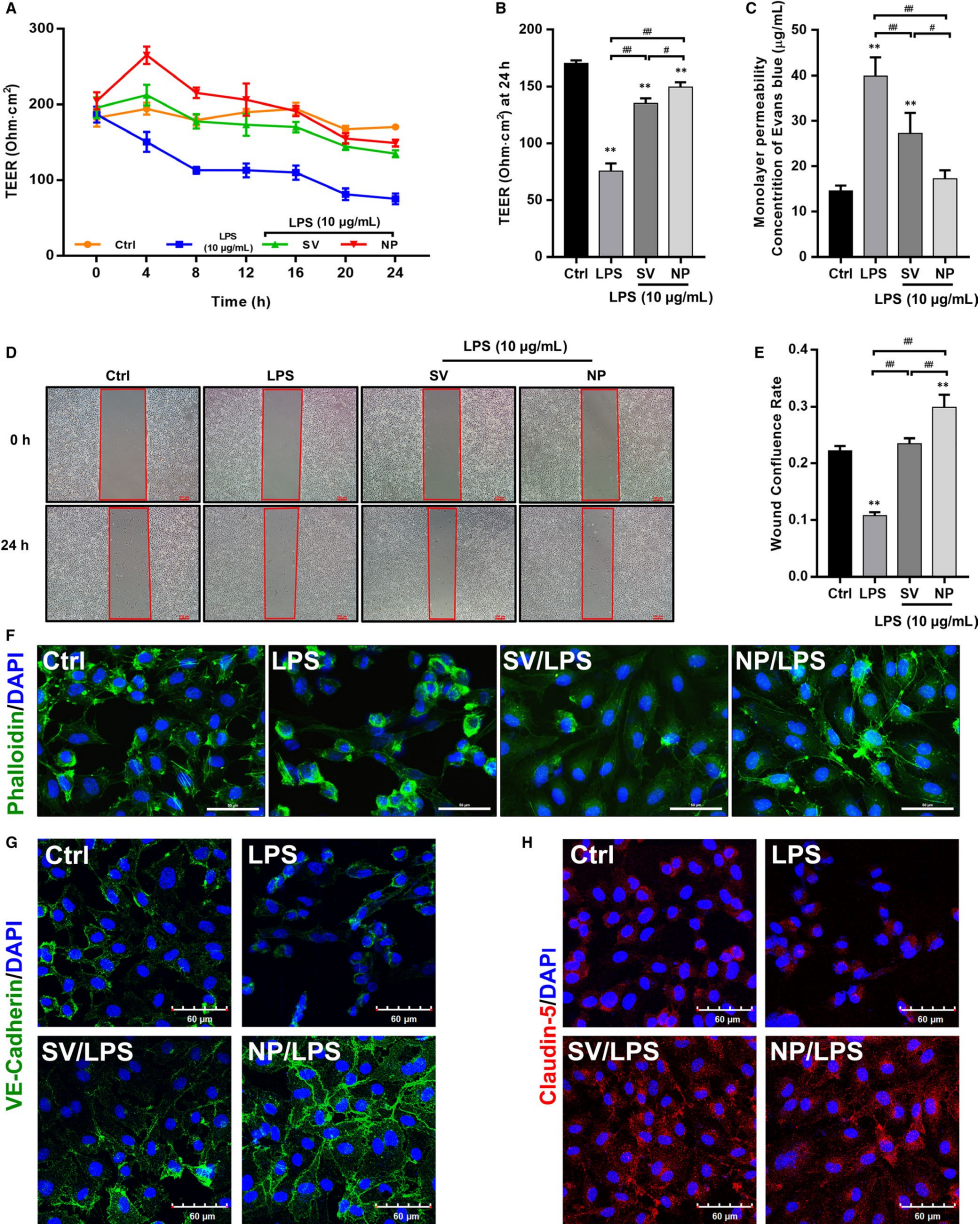
The effects of different SV preparations on TEER, monolayer permeability, cell migration, cytoskeleton and cell junctions of LPS‐treated HUVECs. (A) The TEER of HUVECs after treatment with LPS, normal‐simvastatin (SV) and simvastatin nanoparticle (SV‐NP) at multiple time‐points. (B) The TEER at 24 hours was significantly decreased in LPS group compared with control group and SV/SV‐NP groups (all *P* < .01), the TEER in SV‐NP group was higher than that in SV group (*P* < .05). (C) Monolayer permeability was measured using a Transwell‐Evans Blue (EB) assay. EB concentrations in the lower chambers were increased in LPS group compared with control group and SV/SV‐NP groups (all *P* < .01), and the EB concentrations in SV‐NP group was lower than that in SV group (*P* < .05). (D, E) Quantification of confluence rate at 24 hours which represent the migration ability [% wound confluence = (*a* − *b*) × 100%/*a*; *a* = Initial scratch wound area at 0 hour, *b* = Scratch wound area at 24 hours]. Compared with that in the control group, cell confluence was significantly decreased in the LPS group (*P* < .01) and increased in SV and SV‐NP groups compared with LPS group (all *P* < .01), and the confluence rate in the SV‐NP group was higher than that in the SV group (*P* < .01). (F) FITC‐phalloidin staining showed the cytoskeletal changes in HUVECs injured by LPS in the presence or absence of simvastatin preparations. Green represents phalloidin, and blue indicates nuclei. Scale bar: 50 μm. (G, H) Expression of intercellular junctions (VE‐cadherin and claudin‐5) was evaluated by immunofluorescence labelling. Green represents VE‐cadherin, red indicates claudin‐5, and blue indicates nuclei. Scale bar: 60 μm. Data are from three independent experiments, and error bars represent the standard deviation, **P < .01 vs control group, ^#^ *P* < .05, ^##^ *P* < .01

We evaluated the endothelial barrier function by determining HUVEC monolayer permeability and integrity, which were estimated from Transwell‐Evans Blue (EB) leakage assays and the transendothelial electric resistance (TEER). As expected, a significant decrease in TEER was observed in the LPS groups with increasing stimulation times, while the decrease was restored after simvastatin intervention (Figure 1A). Similar results were also found in human aortic endothelial cells (HAECs) and human pulmonary microvascular endothelial cells (HPMECs) (Figure S3A,B). The average TEER level of the LPS group at 24 hours was lower than that of the control, SV and SV‐NP groups, while the average TEER at 24 hours of the SV‐NP group was higher than that of the SV group (Figure 1B). The leakage of EB from the upper chamber in the LPS group, representing monolayer permeability, was increased compared with the control, SV and SV‐NP groups. Furthermore, EB leakage in the SV‐NP group was lower than that in the SV group (Figure 1C). To determine the effects of different formulations of SV on HUVEC migration after treatment with LPS, cell migration rates were measured using a wound healing assay (Figure 1D,E). The results show that the rates of scratch wound confluence after 24 hours in the LPS group were significantly decreased compared with the control, SV and SV‐NP groups, and the confluence rates of the SV‐NP group were higher than those of the SV group. SV and SV‐NP also recovered confluence rates of HAECs and HPMECs treated with LPS (Figure S3C,E). Immunofluorescence staining with fluorescein isothiocyanate (FITC)‐phalloidin showed that the actin fibres in HUVECs, HAECs and HPMECs were clearly visible and regularly arranged under the cell membrane in the control group. Obvious skeletal remodelling (disorderly arrangement, different thicknesses, unevenly distributed microfilaments and missing cytoskeleton) was observed in the LPS groups. The cytoskeletal structure was partially restored after treatment with different SV preparations, and the effect of the SV‐NP was more significant than that of SV (Figures 1F and S3D,F). Immunofluorescence staining showed that the fluorescence intensities of VE‐cadherin and claudin‐5, which decreased after LPS treatment, were partially recovered after treatment with different SV preparations (Figure 1G,H), and the recovery of VE‐cadherin in the SV‐NP group was more significant than that in SV group (Figure 1G), while there was no significant difference in claudin‐5 fluorescence intensities and protein expression between SV and SV‐NP groups (Figures 1H and S4C,D).

### 3.2 The effects of SV preparations on the expression of IQGAP1 protein, activity of the PDGFRβ/PI3K/Akt signalling pathway and PDGF‐BB in supernatants of cultures of LPS‐treated HUVECs

**Figure 2:**
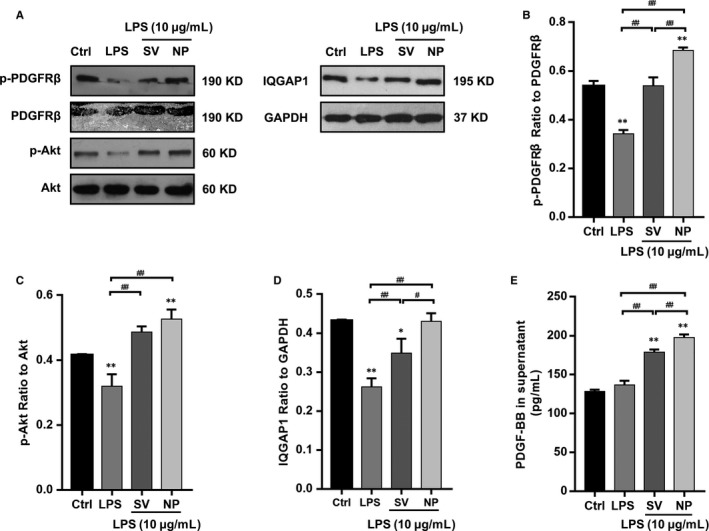
The effects of SV preparations on PDGFRβ phosphorylation, Akt phosphorylation, IQGAP1 expression and PDGF‐BB levels in supernatants of HUVECs cultures treated with LPS. (A‐C) The ratios of p‐PDGFRβ to total PDGFRβ protein and p‐Akt to total Akt protein were remarkably decreased in the LPS group compared with control group and SV/SV‐NP groups (all *P* < .01), the ratio of p‐PDGFRβ to total PDGFRβ in SV‐NP group was higher than that in SV group (*P* < .01), and there is no significant difference in Akt phosphorylation between SV and SV‐NP groups. (A, D) The relative protein levels are expressed as the ratio of the target protein to glyceraldehyde 3‐phosphate dehydrogenase (GAPDH), the levels of IQGAP1 were remarkably down‐regulated in the LPS group compared with control group and SV/SV‐NP groups (all *P* < .01), and the IQGAP1 protein level in SV‐NP group was higher than that in SV group (*P* < .05). (E) The levels of PDGF‐BB in supernatants of the cultures were increased in the SV and SV‐NP groups compared with LPS group (all *P* < .01), and the PDGF‐BB level was higher in SV‐NP group than SV group (*P* < .01). *P < .05 vs control group, **P < .01 vs control group, ^#^ *P* < .05, ^##^ *P* < .01

IQGAP1 protein expression was quantified by Western blotting. The results show that the expression of IQGAP1 was decreased in the LPS group compared with the control, SV and SV‐NP groups, and the level of IQGAP1 protein expression in the SV‐NP group was higher than that in the SV group (Figure 2A,D). The ratios of p‐PDGFRβ to total PDGFRβ protein and of p‐Akt to total Akt protein were remarkably decreased in the LPS group when compared to the control, SV and SV‐NP groups. The ratio of p‐PDGFRβ to total PDGFRβ in the SV‐NP group was higher than that in the SV group (Figure 2A‐C). The levels of PDGF‐BB in culture supernatants were increased in the SV and SV‐NP groups when compared to the LPS group, and the PDGF‐BB level was higher in the SV‐NP group than in the SV group (Figure 2E).

### 3.3 The reparative effect of SV preparations on LPS‐induced endothelial injury was mediated by IQGAP1

**Figure 3:**
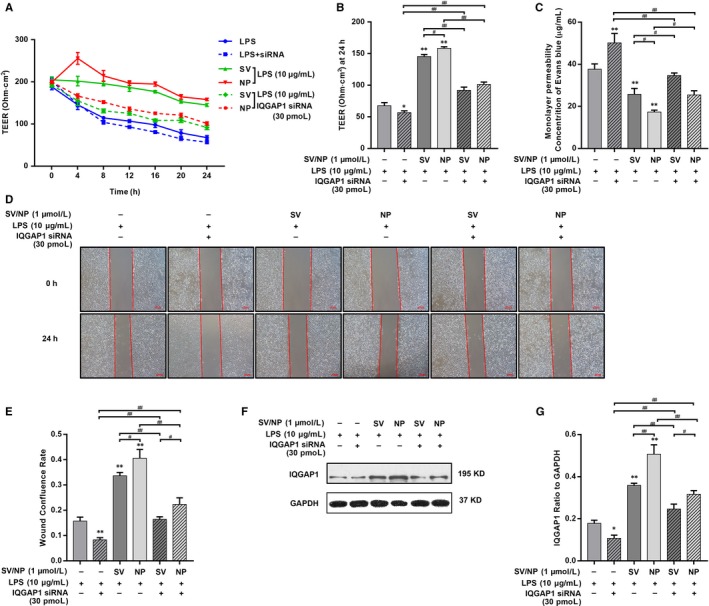
The effects of IQGAP1 knockdown on TEER, monolayer permeability and cell migration of LPS‐injured HUVECs treated with SV preparations. (A) The TEER of different simvastatin preparations treatments on LPS‐injured HUVECs after IQGAP1 knockdown at multiple time‐points. (B) The TEER at 24 hours was significantly decreased in LPS + IQGAP1 siRNA group compared with LPS group (*P* < .05) and decreased in SV/SV‐NP + IQGAP1 siRNA groups compared with SV/SV‐NP groups (all *P* < .01), and the TEER at 24 hours in SV/SV‐NP + IQGAP1 siRNA groups was higher than those in LPS + IQGAP1 siRNA group (all *P* < .01). (C) EB concentrations in the lower chambers were significantly increased in LPS + IQGAP1 siRNA group compared with LPS group (*P* < .01) and increased in SV/SV‐NP + IQGAP1 siRNA groups compared with SV/SV‐NP groups (all *P* < .05), and the EB concentrations in SV/SV‐NP + IQGAP1 siRNA groups were lower than those in LPS + IQGAP1 siRNA group (all *P* < .01). (D, E) Cell confluence rates at 24 hours were significantly decreased in the LPS + IQGAP1 siRNA group compared with the LPS group (*P* < .01) and decreased in SV/SV‐NP + IQGAP1 siRNA groups compared with SV/SV‐NP groups (all *P* < .01), the confluence rates in SV/SV‐NP + IQGAP1 siRNA groups were higher than those in LPS + IQGAP1 siRNA group (all *P* < .01). (F, G) IQGAP1 protein level was down‐regulated in the LPS + IQGAP1 siRNA group compared with the LPS group (*P* < .05) and decreased in SV/SV‐NP + IQGAP1 siRNA groups compared with SV/SV‐NP groups (all *P* < .01), and IQGAP1 protein level in SV/SV‐NP + IQGAP1 siRNA groups was higher than those in LPS + IQGAP1 siRNA group (all *P* < .01). The negative control siRNA has no significant effect on TEER, monolayer permeability, cell migration (Figure S1), PDGFRβ phosphorylation, Akt phosphorylation, IQGAP1 expression and PDGF‐BB in supernatants of the cultures of HUVECs (Figure S2). *P < .05 vs LPS group, **P < .01 vs LPS group, ^#^ *P* < .05, ^##^ *P* < .01

To examine whether different formulations of SV regulated the endothelial barrier function mediated by IQGAP1, HUVECs were transfected with a specific IQGAP1 targeting siRNA, which knockdown efficiency was about 70% (Figure S4B). The knockdown of endogenous IQGAP1 inhibited SV‐induced barrier protection significantly. IQGAP1 siRNA countered the restoration of TEER after SV intervention, and the TEER at 24 hours was significantly decreased in the SV/SV‐NP + IQGAP1 siRNA groups compared with the SV/SV‐NP groups and increased in the SV/SV‐NP + IQGAP1 siRNA groups when compared to the LPS + IQGAP1 siRNA group (Figure 3A,B). The leakage of EB from the upper chamber in each group showed the opposite trend as the TEER levels (Figure 3C). The cell confluence rates at 24 hours were significantly decreased in the LPS + IQGAP1 siRNA group compared with the LPS group and significantly decreased in the SV/SV‐NP + IQGAP1 siRNA groups compared with the SV/SV‐NP groups. The confluence rates in SV/SV‐NP + IQGAP1 siRNA groups were higher than those in the LPS + IQGAP1 siRNA group (Figure 3D,E). Western blotting showed that the expression of IQGAP1 protein in the HUVECs that contained IQGAP1 siRNA was significantly lower than the level in cells without IQGAP1 siRNA (Figure 3F,G). The negative control siRNA has no significant effect on barrier function and related signalling pathway (Figures S1 and S2)

### 3.4 The reparative effect of different SV preparations on LPS‐induced endothelial injury was mediated by the PI3K/Akt pathway

**Figure 4:**
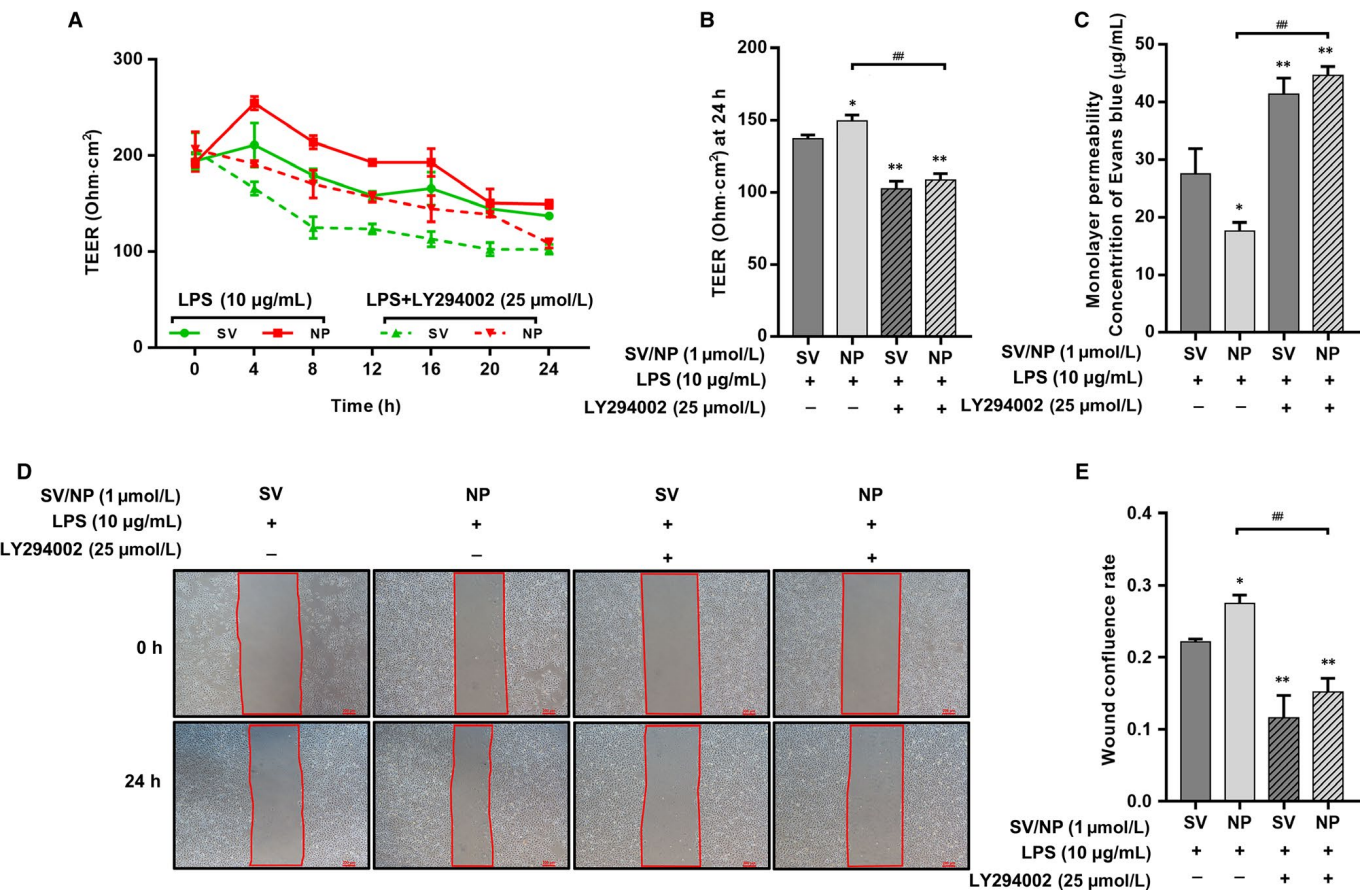
The effects of PI3K/Akt inhibition on TEER, monolayer permeability and cell migration of LPS‐injured HUVECs treated with SV preparations. (A) The TEER of different simvastatin preparations treatments on LPS‐injured HUVECs after PI3K/Akt inhibition at multiple time‐points. (B) The TEER at 24 hours was significantly decreased in the SV/SV‐NP + LY294002 groups compared with the SV/SV‐NP groups (all *P* < .01). (C) EB concentrations in the lower chambers were increased in SV/SV‐NP + LY294002 groups compared with SV/SV‐NP groups (all *P* < .01). (D) Cell confluence rates at 24 hours were significantly decreased in the SV/SV‐NP + LY294002 groups compared with the SV/SV‐NP groups (all *P* < .01). The LY294002 has no significant effect on TEER, monolayer permeability and cell migration of HUVECs (Figure S1). *P < .05 vs SV + LPS group, **P < .01 vs SV + LPS group, ^##^ *P* < .01

**Figure 5:**
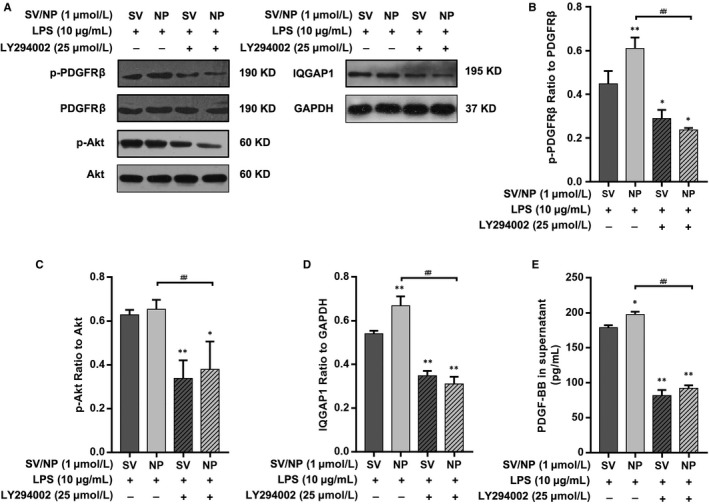
The effects of PI3K/Akt inhibition on PDGFRβ phosphorylation, IQGAP1 expression and PDGF‐BB in supernatants of cultures of LPS‐injured HUVECs treated with SV preparations. (A, B) The ratio of p‐PDGFRβ to total PDGFRβ protein was remarkably decreased in the SV/SV‐NP + LY294002 groups compared with the SV/SV‐NP groups (SV + LY294002: *P* < .05, SV‐NP + LY294002: *P* < .01). (A, C) The ratio of p‐Akt to total Akt protein was remarkably decreased in the SV/SV‐NP + LY294002 groups compared with the SV/SV‐NP groups (all *P* < .01). (A, D, E) The IQGAP1 protein level and PDGF‐BB in supernatants of the cultures in the SV/SV‐NP + LY294002 groups were remarkably down‐regulated than those in the SV/SV‐NP groups (all *P* < .01). The LY294002 has no significant effect on PDGFRβ phosphorylation, Akt phosphorylation, IQGAP1 expression and PDGF‐BB in supernatants of the cultures of HUVECs (Figure S2). *P < .05 vs SV + LPS group, **P < .01 vs SV + LPS group, ^##^ *P* < .01

We next used LY294002 (25 μmol/L), a PI3K inhibitor, to confirm whether inhibition of the PI3K/Akt pathway inhibited the protective effect of SV preparations. LY294002 countered the restoration of TEER after SV intervention with increasing stimulation times (Figure 4A). The average TEER at 24 hours in the LY294002 + SV/SV‐NP groups were significantly decreased compared with the SV/SV‐NP groups (Figure 4B). A Transwell‐EB monolayer permeability assay showed that the leakage of EB in the LY294002+SV/SV‐NP groups was significantly higher than those in the SV/SV‐NP groups (Figure 4C). The cell confluence rate at 24 hours in the LY294002 + SV/SV‐NP groups was decreased compared with those in the SV/SV‐NP groups (Figure 4D,E). In a similar manner, Western blotting and enzyme‐linked immunosorbent assays (ELISAs) showed that IQGAP1 expression and PDGF‐BB in supernatants of the cultures from the SV/SV‐NP + LY294002 groups were remarkably down‐regulated compared with the SV/SV‐NP groups (Figure 5A,D,E). The ratio of phosphorylated PDGFRβ to PDGFRβ protein in the LY294002 + SV/SV‐NP groups, reflecting the degree of PDGFRβ activation, was significantly decreased compared with the SV/SV‐NP groups (Figure 5A,B). Phosphorylated Akt protein levels were also inhibited by LY294002 (Figure 5A,C).

### 3.5 The reparative effect of different SV preparations on LPS‐induced endothelial injury was mediated by PDGFRβ/PDGF‐BB

**Figure 6:**
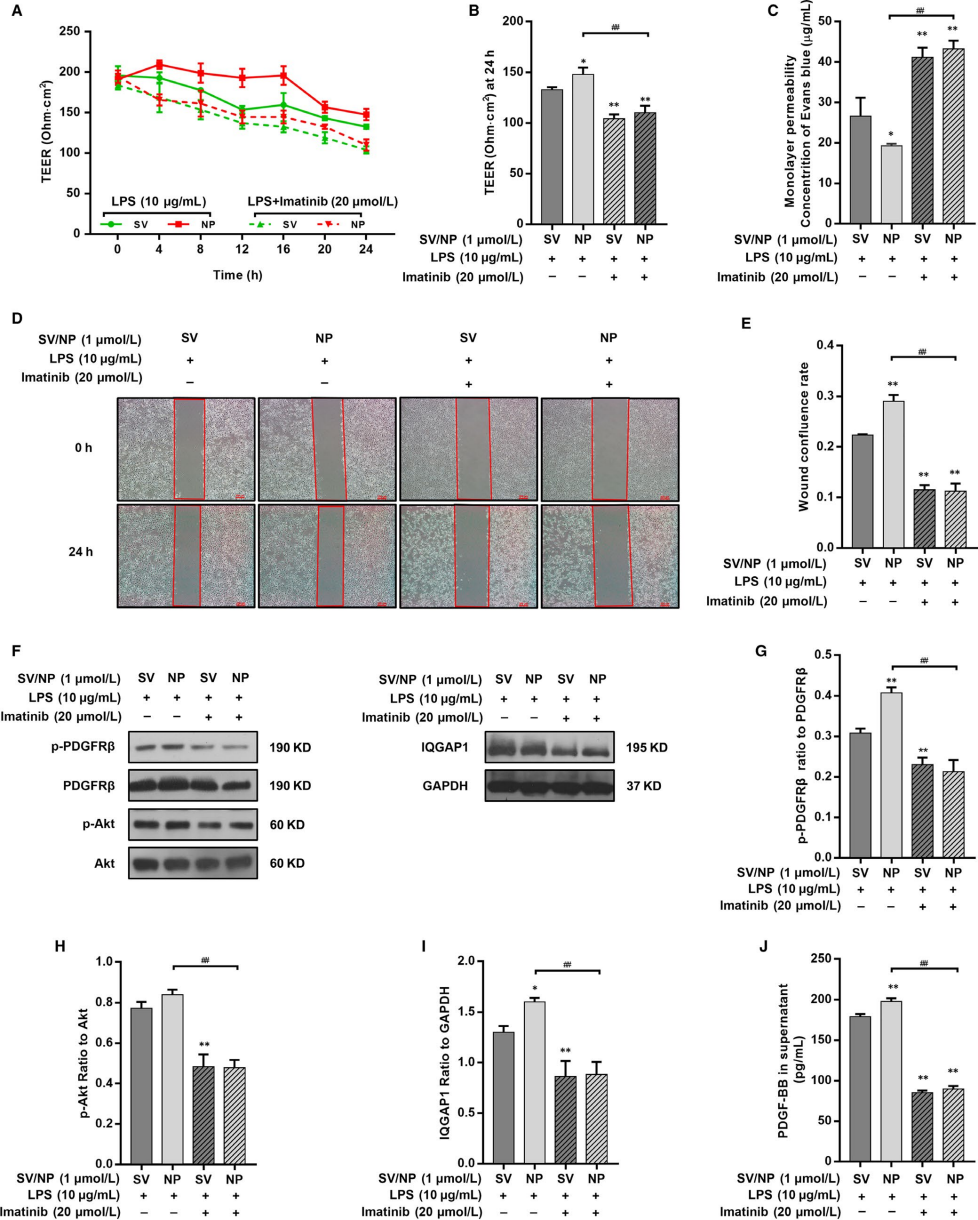
The effects of PDGFRβ inhibition on TEER, monolayer permeability, cell migration and the PDGFRβ/PI3K/Akt/IQGAP1 pathway activity of LPS‐injured HUVECs treated with SV preparations. (A) The TEER of different simvastatin preparations treatments on LPS‐injured HUVECs after PDGFR inhibition at multiple time‐points. (B) The TEER at 24 hours was significantly decreased in the SV/SV‐NP + Imatinib groups compared with the SV/SV‐NP groups (all *P* < .01). (C) EB concentrations in the lower chambers were increased in SV/SV‐NP + Imatinib groups compared with SV/SV‐NP groups (all *P* < .01). (D, E) Cell confluence rates at 24 hours were significantly decreased in the SV/SV‐NP + Imatinib groups compared to the SV/SV‐NP groups (all *P* < .01). (F‐H) The ratios of p‐PDGFRβ to total PDGFRβ protein and p‐Akt to total Akt protein were remarkably decreased in the SV/SV‐NP + Imatinib groups compared with the SV/SV‐NP groups (all *P* < .01). (F, I, J) IQGAP1 protein level and PDGF‐BB in supernatants of the cultures were remarkably decreased in the SV/SV‐NP + Imatinib groups compared with the SV/SV‐NP groups (all *P* < .01). The Imatinib has no significant effect on TEER, monolayer permeability, cell migration and PDGFRβ/PI3K/Akt/IQGAP1 pathway activity of HUVECs (Figure S1, 2). *P < .05 vs SV + LPS group, **P < .01 vs SV + LPS group, ^##^ *P* < .01

Based on our Western blotting and cytotoxicity titration assay results (Figure S4A), we used 20 μmol/L imatinib, an inhibitor of PFGFRβ, to investigate the changes in barrier protection by SV in the presence or absence of PDGFRβ phosphorylation. Both the Transwell‐EB monolayer permeability assay and TEER measurements showed that the barrier function of the SV/SV‐NP + imatinib groups was significantly decreased compared with those of the SV/SV‐NP groups (Figure 6A‐C). A wound healing assay showed that the cell migration capacity of the SV/SV‐NP + imatinib groups was decreased compared with those of the SV/SV‐NP groups (Figure 6D,E). Western blot analysis showed that the level of IQGAP1 protein in SV/SV‐NP + imatinib groups was significantly down‐regulated compared with those of SV/SV‐NP groups (Figure 6F,I). Similarly, Western blotting and ELISAs showed that the ratio of phosphorylated Akt to Akt protein and PDGF‐BB in supernatants of cultures in the SV/SV‐NP + imatinib groups was remarkably down‐regulated compared with the SV/SV‐NP groups (Figure 6F,H,J). The ratio of phosphorylated PDGFRβ to PDGFRβ was also inhibited by imatinib (Figure 6F,G). Both inhibitors independently did not affect TEER, monolayer permeability and cell migration of HUVECs (Figure S1A‐D) and, more importantly, did not affect PDGFRβ phosphorylation, Akt phosphorylation, IQGAP1 expression and PDGF‐BB in supernatants of the cultures of HUVECs (Figure S2A,B,F),

### 3.6 Different SV preparations promoted the association of IQGAP1 with PDGFRβ

**Figure 7:**
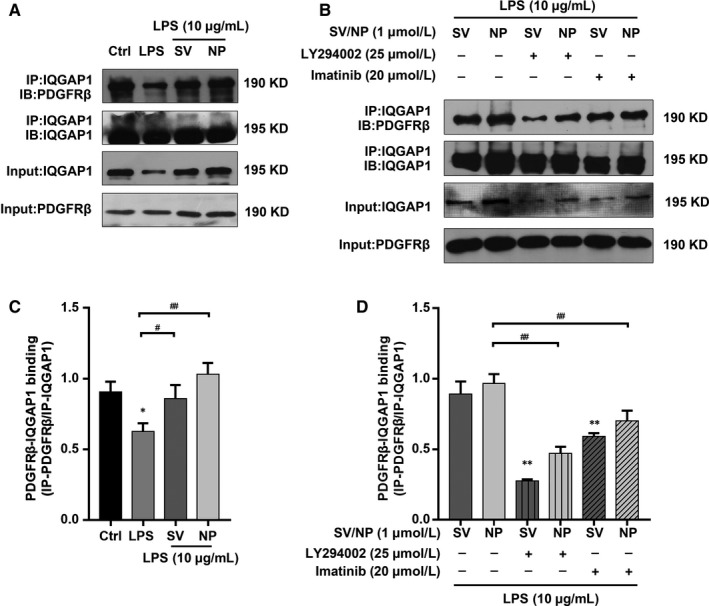
The effects of SV preparations on the PDGFRβ/IQGAP1 binding ability of HUVECs treated with LPS and the effects of PDGFRβ/PI3K/Akt inhibition on the PDGFRβ/IQGAP1‐binding ability of LPS‐injured HUVECs treated with SV preparations. (A, C) Detection of PDGFRβ/IQGAP1 physical association by immunoprecipitation. The PDGFRβ/IQGAP1 binding was remarkably decreased in LPS group compared with control group (*P* < .05) and SV/SV‐NP groups (SV: *P* < .05, SV‐NP: *P* < .01). (B, D) PDGFRβ/IQGAP1 binding ability was significantly decreased in LY294002 + SV/SV‐NP groups and Imatinib + SV/SV‐NP groups compared with the SV/SV‐NP groups (all *P* < .01). The negative control siRNA, LY294002 and imatinib have no significant effect on PDGFRβ/IQGAP1 binding. Normal IgG was used as a negative control (Figure S2). *P < .05 vs control/SV group, **P < .01 vs control/SV group, ^#^ *P* < .05, ^##^ *P* < .01

To investigate the relationship between IQGAP1 and PDGFRβ in HUVECs of different groups, we examined whether IQGAP1 bound to PDGFRβ and whether two formulations of SV affected their binding. Co‐immunoprecipitation showed that LPS inhibited IQGAP1 binding to PDGFRβ; however, SV/SV‐NP restored and promoted this process (Figure 7A,C). Both LY294002 and imatinib decreased the binding of IQGAP1 and PDGFRβ promoted by SV/SV‐NP (Figure 7B,D). Normal IgG was used as a negative control (Figure S2C), and both inhibitors independently did not affect PDGFRβ/IQGAP1 binding (Figure S2D,E).

## 4 DISCUSSION

Sepsis is a major clinical problem in intensive care units. It is associated with a high mortality rate, and effective therapeutic options are limited; thus, it is important to understand the pathophysiology of sepsis and its related complications. Furthermore, new therapeutic targets are urgently needed. Statins, hydroxymethylglutaryl‐CoA reductase inhibitors, may have effects on inflammatory diseases such as sepsis and ARDS via its multiple properties, including an anti‐inflammatory effect and inhibition of leucocyte adhesion.^35^, ^36^ Statins have become a controversial treatment for sepsis and infectious diseases in recent years.^36^, ^37^, ^38^, ^39^, ^40^ Due to the poor water solubility and strong fat solubility of statins, NP carrier systems have been designed to enhance drug delivery,^41^ and it has been confirmed that SV‐NPs have good efficacy towards chronic inflammatory disorders such as atherosclerosis and chronic pulmonary disease.^29^, ^42^ The formulation of SV‐NPs improves the pharmacokinetic profile and bioavailability of SV^43^; it was also reported that nanostructured lipid carriers, especially those associated with SV, were able to reduce cytotoxicity. A study based on the HUVEC model also showed that SV‐NPs and the associated targeted drugs could ameliorate endothelial dysfunction.^28^ Similarly, our study shows that the barrier protection of SV/SV‐NPs was significant. High‐permeability induced by LPS was successfully reversed by SV preparations, and the effect of SV‐NPs was better than that of SV. We observed a similar phenomenon in human aortic endothelial cells and human pulmonary microvascular endothelial cells (Figure S3). However, it is important to clarify the mechanism of the endothelial barrier protection of SV/SV‐NPs for sepsis.

It has been reported that the regulation of endothelial barrier functions (eg actin dynamics and the cytoskeleton) is a key pathological mechanism in sepsis.^44^ Our study showed obvious cytoskeletal remodelling in HUVECs after LPS injury, and the cytoskeletal structure was partially restored after treatment with different SV preparations. The results of our study indicate that two formulations of SV improved the decreased migratory capacity of LPS‐treated HUVECs and that the effect of SV‐NPs was superior to that of SV. Similar to the results of Zitta,^45^ a weak migratory capacity was also related to high‐permeability in HUVECs, and we found that the permeability of the SV and SV‐NP groups was significantly lower than that of the LPS group.

It is well‐known that IQGAP1 plays an important role in cytoskeletal regulation, especially cell migration. Previous studies reported that the up‐regulation of IQGAP1 expression promoted the adhesion and migration of vascular smooth muscle cells (VSMCs).^16^ In addition, our study showed that cell junctions involving VE‐cadherin and claudin‐5 are altered after IQGAP1 treatment during the processes of endothelial cell injury to recovery. Furthermore, previous studies showed that IQGAP1 mediates barrier function changes by regulating the tight junctions and adherens junctions of endothelial cells.^46^, ^47^, ^48^, ^49^ Some studies reported that after the knockdown of IQGAP1, the morphology and function of vascular endothelial cells were altered, indicating that a decrease in IQGAP1 was the cause of endothelial barrier dysfunction.^11^ Similarly, we found that SV/SV‐NPs up‐regulated the expression of IQGAP1. To investigate the function of IQGAP1 in the regulation of the endothelial barrier, we knocked down IQGAP1 protein expression with a specific siRNA. The results showed that SV/SV‐NP‐induced barrier restoration was significantly inhibited, indicating that IQGAP1 was necessary for SV/SV‐NPs‐mediated endothelial barrier protection. Other studies confirmed that angiopoietin‐1 and sphingosine 1‐phosphate were required for IQGAP1 to induce barrier protection and inhibit microvascular endothelium leakage.^6^, ^11^

It is important to identify the upstream regulatory molecules associated with IQGAP1. According to some studies, the PI3K/Akt pathway is not only a crucial regulatory molecule for various cellular functions, but also important in transduction signalling underlying endothelial barrier function during sepsis.^50^, ^51^, ^52^, ^53^ In the present study, we found that SV/SV‐NPs activated the PI3K/Akt pathway and promoted Akt phosphorylation, which was inhibited by LPS. We then used the PI3K inhibitor LY294002 to determine whether the PI3K/Akt pathway had a mediating function in SV/SV‐NPs‐induced barrier protection. The inhibition of PI3K/Akt signalling suppressed cell migration and barrier restoration induced by SV/SV‐NPs, suggesting that PI3K/Akt signalling was a key regulator in SV/SV‐NPs‐mediated endothelial barrier protection. It has been reported that PDGFRβ is the key controlling component that mediates PI3K/Akt phosphorylation.^54^, ^55^ It was further reported that PDGFRβ is involved with vascular repair in the early phases after vascular injury.^18^, ^56^ Similar to these studies, we found that SV/SV‐NPs promoted PDGFRβ phosphorylation inhibited by LPS, and that imatinib, a PDGFRβ inhibitor, suppressed barrier restoration induced by SV/SV‐NPs. Taken together, our results suggest that the PDGFRβ/PI3K/Akt/IQGAP1 pathway is an important regulator in the recovery process induced by LPS injury and therefore plays a critical role in vascular injury during sepsis.

Platelet‐derived growth factor‐BB plays an important role in many physiological and pathological processes,^57^, ^58^, ^59^, ^60^ including the repair process after tissue injury.^60^, ^61^ In the present study, we found that SV/SV‐NPs promoted PDGF‐BB secretion and that imatinib down‐regulated the increasing PDGF‐BB secretion induced by SV/SV‐NPs. Earlier studies also showed that statins enhanced the expression of PDGFR and PDGF,^62^ so we believe that SV/SV‐NPs may up‐regulate PDGF‐BB secretion in the recovery of barrier functions. A previous study reported that IQGAP1 bound directly to vascular endothelial growth factor receptor‐2 (VEGFR2) to transmit the VEGFR2 signal and promote endothelial cell migration.^15^ However, the role of IQGAP1 in PDGFR signalling in HUVECs is still relatively unexplored. According to some reports, PDGF promotes the association with PDGFRβ and promotes activation of PDGFR in VSMCs.^16^, ^63^

There are some similarities in our studies to these previous reports. We showed that IQGAP1 bound with PDGFRβ in HUVECS and that SV/SV‐NPs restored the association of IQGAP1 and PDGFRβ suppressed by LPS during vascular injury. We hypothesized that SV/SV‐NPs regulated the association of the two molecules via the PDGFRβ/PI3K/Akt pathway, and we subsequently showed that the binding of IQGAP1 and PDGFRβ was inhibited by treatment with LY294002 and imatinib. These findings suggest that the PDGFRβ/PI3K/Akt signalling pathway was involved in the binding ability of IQGAP1 and PDGFRβ regulated by SV/SV‐NPs in vitro. Whether SV/SV‐NPs have protective effects on the barrier function of a sepsis model in vivo must be addressed in our future studies.

In summary, the present study shows that barrier protection by SV/SV‐NPs in LPS‐induced vascular injury was involved in the up‐regulation of IQGAP1 expression, the binding of IQGAP1 and PDGFRβ, and PDGFRβ activation in HUVECs, which promoted cell migration and decreased endothelial permeability, and eventually restored the damaged endothelial barrier. These findings provide novel insight into PDGFRβ/PI3K/Akt/IQGAP1 as a potential therapeutic target for sepsis and its related diseases, including ARDS and AKI.

## CONFLICT OF INTEREST

All authors certify that they have no affiliations with or involvement in any organization or entity with any financial interest, or non‐financial interest in the subject matter or materials discussed in this manuscript.

## AUTHORS' CONTRIBUTIONS

W. Z. wrote the main manuscript text, X. Z. and ZW participated in part of the experiments and analysed the data.

## Data Availability

The data that support the findings of this study are available from the corresponding author upon reasonable request.
