# Supplementary figures and images for: Simvastatin preparations promote PDGF‐BB secretion to repair LPS‐induced endothelial injury through the PDGFRβ/PI3K/Akt/IQGAP1 signalling pathway
Source: J Cell Mol Med. 2019 Oct 1;23(12):8314–27. doi: 10.1111/jcmm.14709 (PMC6850957; doi:10.1111/jcmm.14709)

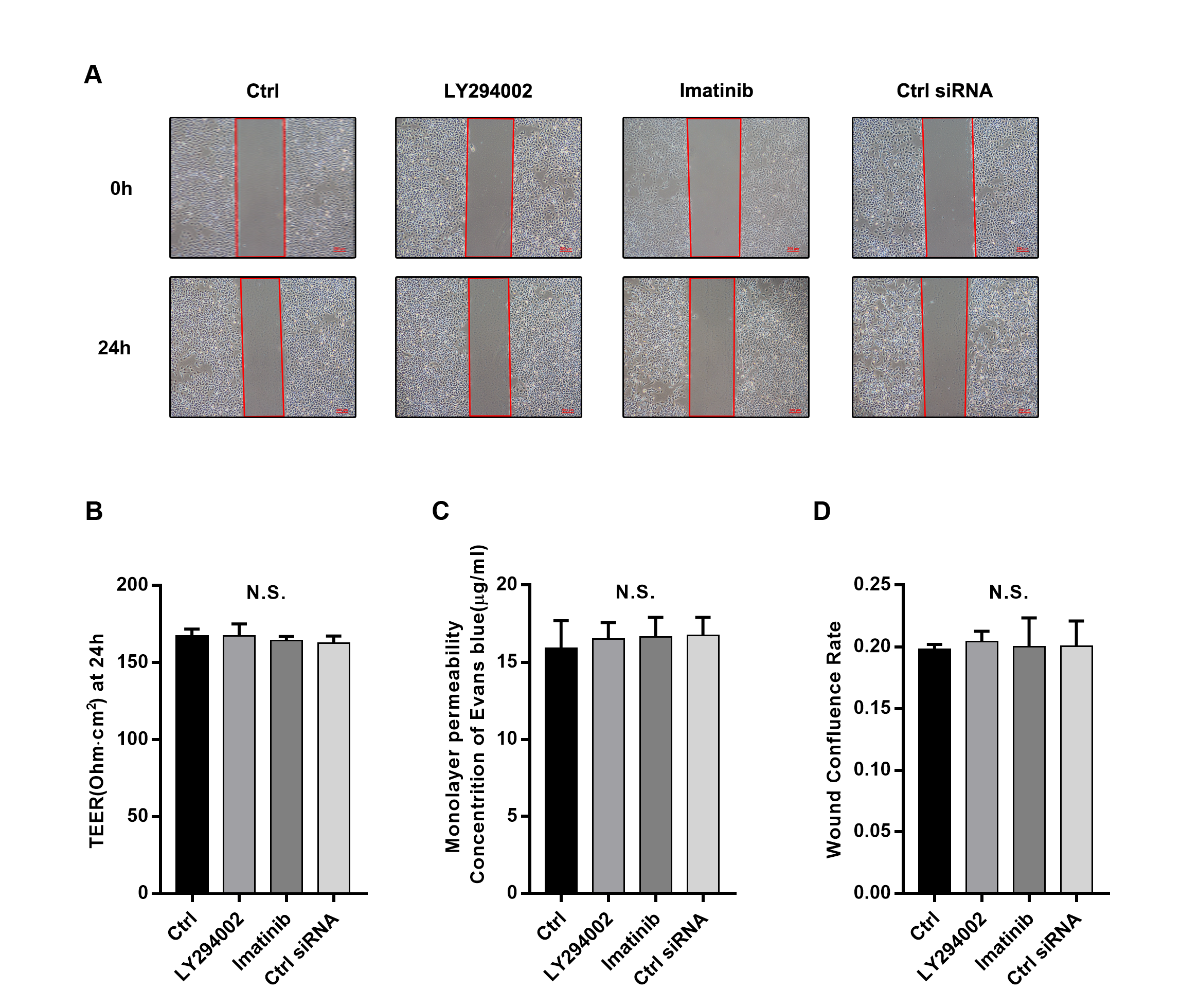

Supplement: Supplementary file 1 [file JCMM-23-8314-s001.tif]

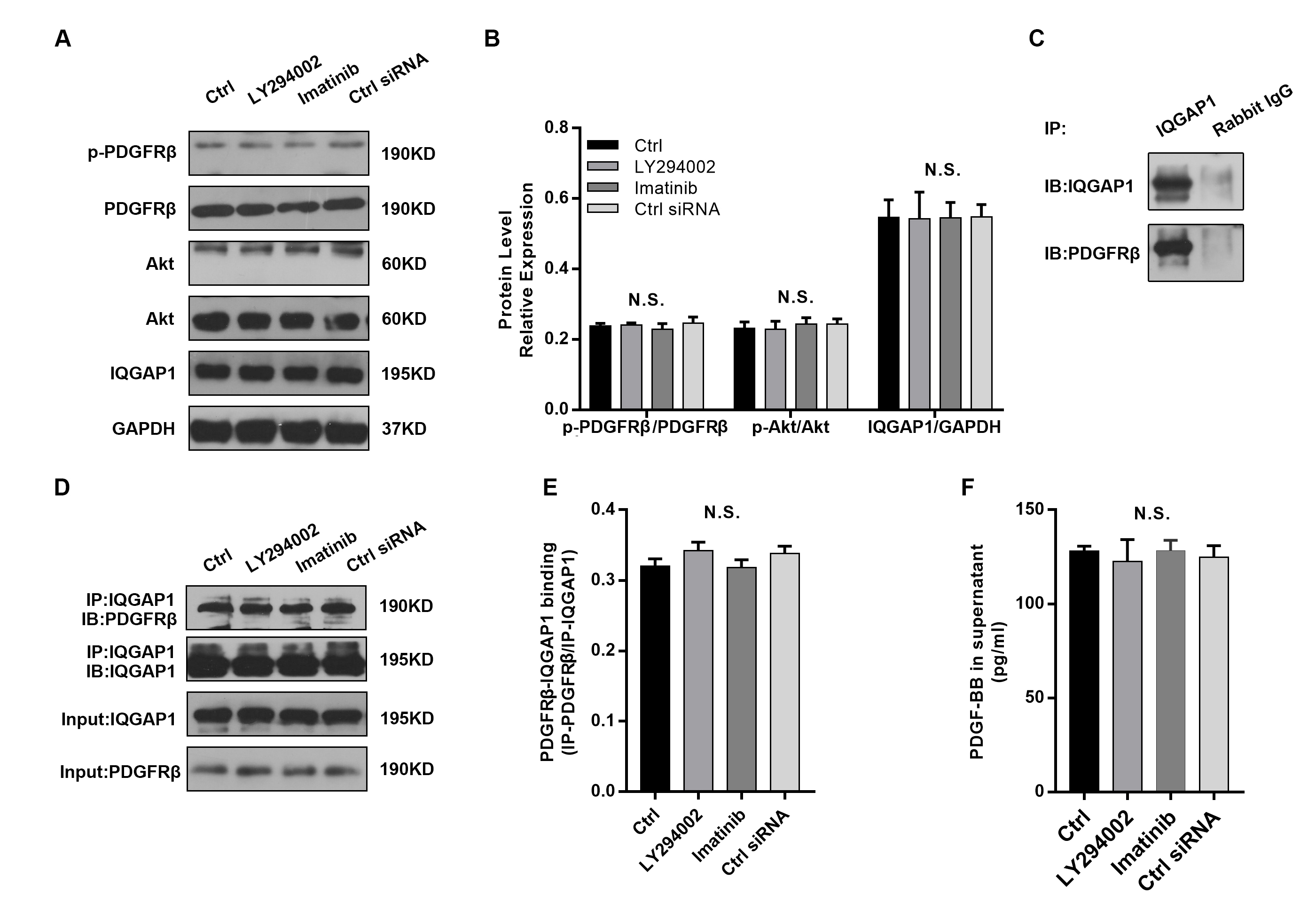

Supplement: Supplementary file 2 [file JCMM-23-8314-s002.tif]

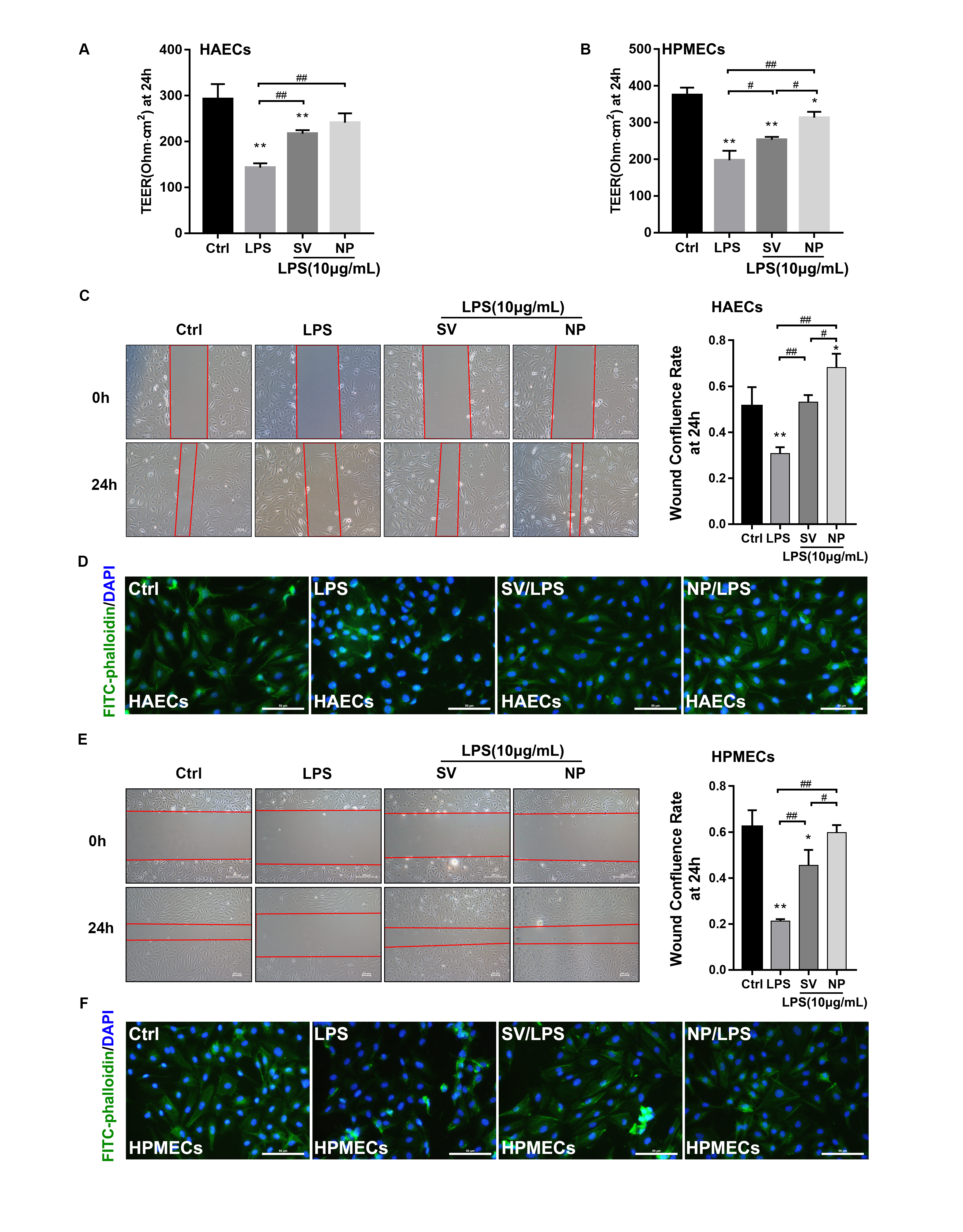

Supplement: Supplementary file 3 [file JCMM-23-8314-s003.tif]

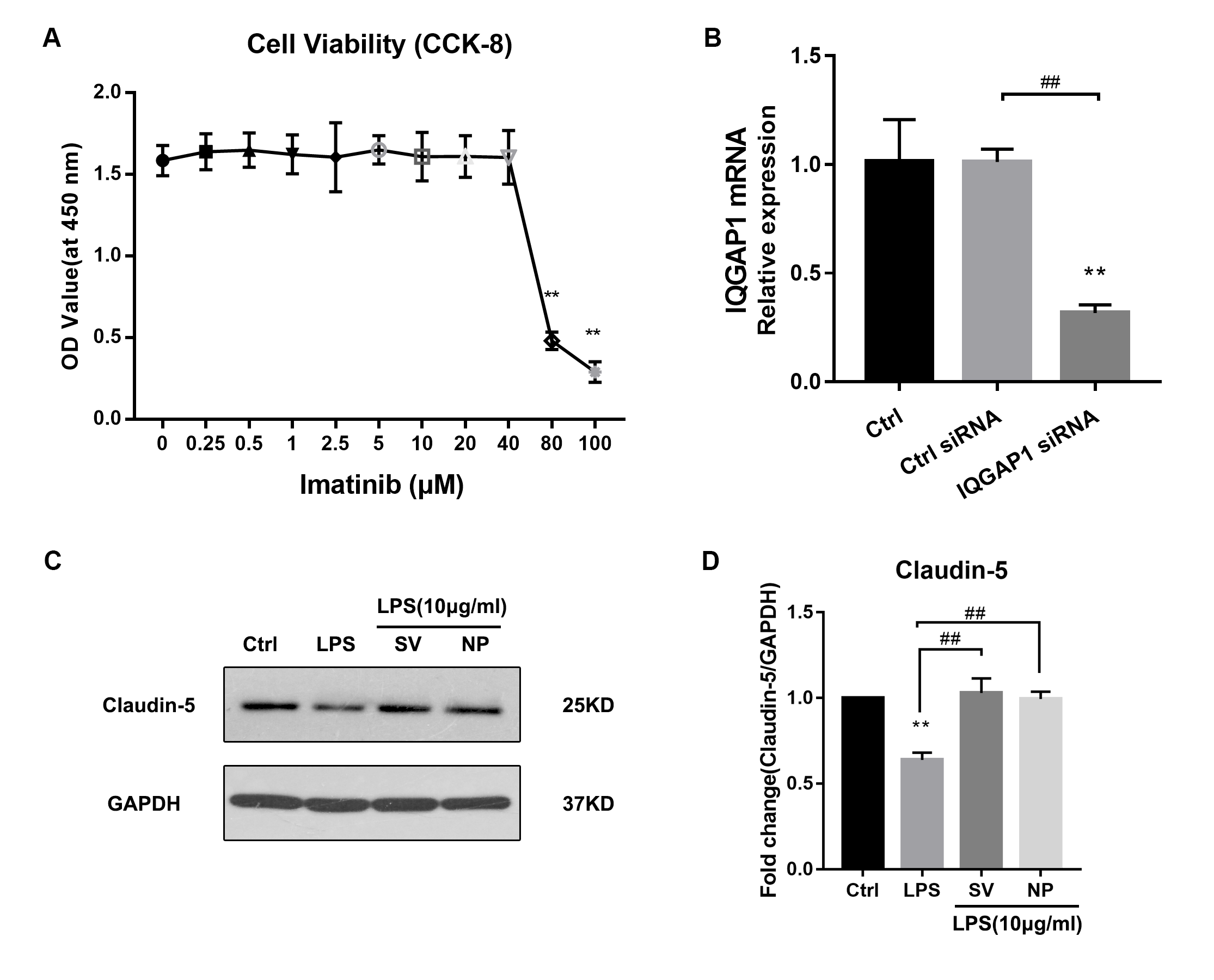

Supplement: Supplementary file 4 [file JCMM-23-8314-s004.tif]
